# Dose deviations induced by respiratory motion for radiotherapy of lung tumors: Impact of CT reconstruction, plan complexity, and fraction size

**DOI:** 10.1002/acm2.12847

**Published:** 2020-03-12

**Authors:** Erlend P. S. Sande, Ana M. Acosta Roa, Taran P. Hellebust

**Affiliations:** ^1^ Department of Medical Physics Oslo University Hospital Oslo Norway; ^2^ Department of Physics University of Oslo Oslo Norway

**Keywords:** complexity, interplay, lung, radiotherapy, SBRT, VMAT

## Abstract

A thorax phantom was used to assess radiotherapy dose deviations induced by respiratory motion of the target volume. Both intensity modulated and static, non‐modulated treatment plans were planned on CT scans of the phantom. The plans were optimized using various CT reconstructions, to investigate whether they had an impact on robustness to target motion during delivery. During irradiation, the target was programmed to simulate respiration‐induced motion of a lung tumor, using both patient‐specific and sinusoidal motion patterns in three dimensions. Dose was measured in the center of the target using an ion chamber. Differences between reference measurements with a stationary target and dynamic measurements were assessed. Possible correlations between plan complexity metrics and measured dose deviations were investigated. The maximum observed motion‐induced dose differences were 7.8% and 4.5% for single 2 Gy and 15 Gy fractions, respectively. The measurements performed with the largest target motion amplitude in the superior–inferior direction yielded the largest dosimetric deviations. For 2 Gy fractionation schemes, the summed dose deviation after 33 fractions is likely to be less than 2%. Measured motion‐induced dose deviations were significantly larger for one CT reconstruction compared to all the others. Static, non‐modulated plans showed superior robustness to target motion during delivery. Moderate correlations between the modulation complexity score applied to VMAT (MCSv) and measured dose deviations were found for 15 Gy SBRT treatment plans. Correlations between other plan complexity metrics and measured dose deviations were not found.

## 1 Introduction

Radiotherapy of lung cancer entails the challenge of delivering a prescribed dose to a target subjected to respiratory motion in a hypo‐dense environment. While delivery techniques like volumetric modulated arc therapy (VMAT) offer superior dose conformity also for lung tumors, they also come with a need for high‐quality image guidance and potentially also motion management.

Several authors have reported on respiration‐induced tumor motion patterns and typical amplitudes of lung tumor displacement.^1^, ^2^, ^3^, ^4^, ^5^ The largest motion amplitudes are found in the superior–inferior (SI) direction, where maximum tumor excursions of 20–30 mm, or even up to more than 50 mm in rare cases, are reported. The mean SI amplitudes are usually found to be below 10 mm. Motion amplitudes in the anterior–posterior (AP) and left–right (LR) directions are, not surprisingly, reported to be significantly smaller. Lower lobe tumors displacements were typically found to be larger than those of tumors elsewhere in the lung.

Dosimetric deviations due to intra‐fractional motion of the target volume should be divided into *blurring effects* and *interplay effects*, as described by several authors.^6^, ^7^, ^8^ The *blurring effect* will occur both for static 3D conformal radiotherapy (CRT) fields and for modulated treatment fields, and may be understood as a “smearing out” of the (more or less) inhomogeneous static dose distribution seen in the treatment planning system (TPS), due to motion. The exact dose to the moving tumor will depend on the nature of the motion, even for non‐modulated fields and even when appropriate margins encompassing the entire tumor excursion are applied. For highly inhomogeneous dose distributions such as stereotactic body radiotherapy (SBRT) plans, where the central gross tumor volume (GTV) dose may be 50% higher than the peripheral planning target volume (PTV) dose, one may imagine that the blurring effect is even more prominent. The *interplay effect* is associated only with modulated fields such as intensity modulated radiotherapy (IMRT) or VMAT plans. These plans are optimized with dynamic multi‐leaf collimator (MLC) motions, resulting in a desired — often complex and inhomogeneous — dose distribution as shown in the TPS. When intra‐fractional motion occurs during delivery, planned MLC motions and apertures do not necessarily coincide with the patient anatomy as represented in the computed tomography (CT) images used for optimization and dose calculation. This, in turn, may lead to under‐ or overdosage of the target or organs at risk (OARs) compared to the predicted dose as shown in the TPS.

Investigations on the impact of motion on dose delivery to lung tumors have been done by previous authors both by simulation and phantom measurements. Several simulation studies.^7^, ^9^, ^10^, ^11^, ^12^ have looked into modulated SBRT treatments. These reported only small dose deviations to the target volume, as long as sufficient margins were applied, and as long as tumor excursions were not too large. One study^13^ described simulation of interplay effects for both high and low doses per fraction (simulation methods were also validated with measurements). Simulated motion‐induced dose deviations of up to 17% for a single fraction were reported. A number of studies have reported on the dosimetric impact of intra‐fraction motion based on *measurements* — for conventionally fractionated as well as hypofractionated treatment plans. Most of these used 1D or 2D sinusoidal motion, while a few reported using more complex motion such as 3D motion, or actual patient respiration curves. Reported motion amplitudes were in the 6–30 mm range. Both irradiation of phantoms with IMRT plans^14^, ^15^, ^16^, ^17^ and VMAT plans — or a combination of VMAT and other delivery techniques.^18^, ^19^, ^21^, ^29^ — have been investigated. The range of reported motion‐induced dose deviations — that is, measurements during dynamic delivery compared to measurements during stationary delivery — is quite wide. Dose deviations during non‐gated delivery of up to 30%, 2–18%, and 2–5% are reported for one single field, one fraction delivered with multiple fields, and a series of 8–30 fractions, respectively.

Various authors have investigated possible correlations between plan complexity metrics and dosimetric accuracy.^23^, ^24^, ^25^, ^26^, ^27^ Various measuring devices, delivery techniques, and complexity metrics were used. Results were not unambiguous, as some papers reported significant correlations, and some did not.

In radiotherapy planning, there are several options when choosing a CT image set for dose calculation. Some image reconstruction techniques and resulting image datasets may be more representative to the patient anatomy at treatment, compared to others. Other image sets may be superior in terms of accurate dosimetry, when measurements are compared to TPS calculation.^27^ The various CT reconstructions as a basis for dose calculation are to some degree shown to be equivalent in terms of dosimetric accuracy and anatomical representation.^28^, ^29^, ^30^ In the current study, we seek to determine whether optimizing and calculating plans based on different CT reconstructions has an impact on plan robustness, in terms of dosimetric deviations due to respiration‐induced target motion during treatment. Relative dose deviations will be obtained by comparing dynamic measurements with stationary measurements, thereby isolating interplay and blurring effects from dosimetric agreement with TPS calculated dose. Furthermore, we seek to quantify possible interplay effects using different fractionation schemes, various motion amplitudes, and various respiration patterns. Possible correlations between measured dose deviations and various plan complexity metrics will be investigated. CT scanning procedures, contouring, and treatment planning comply with our institution’s standard practice.

The novelty of the current study is twofold. To our knowledge, previous works have not investigated the possible impact that optimizing plans based on different CT reconstructions may have on robustness to target motion, in terms of measured interplay effects. Furthermore, previous works have not, to our knowledge, investigated possible correlations between VMAT complexity metrics and measured motion‐induced dose deviations for highly hypofractionated as well as normofractionated lung treatments.

## 2 Methods and materials

### 2.1 CIRS phantom

**Fig. 1:**
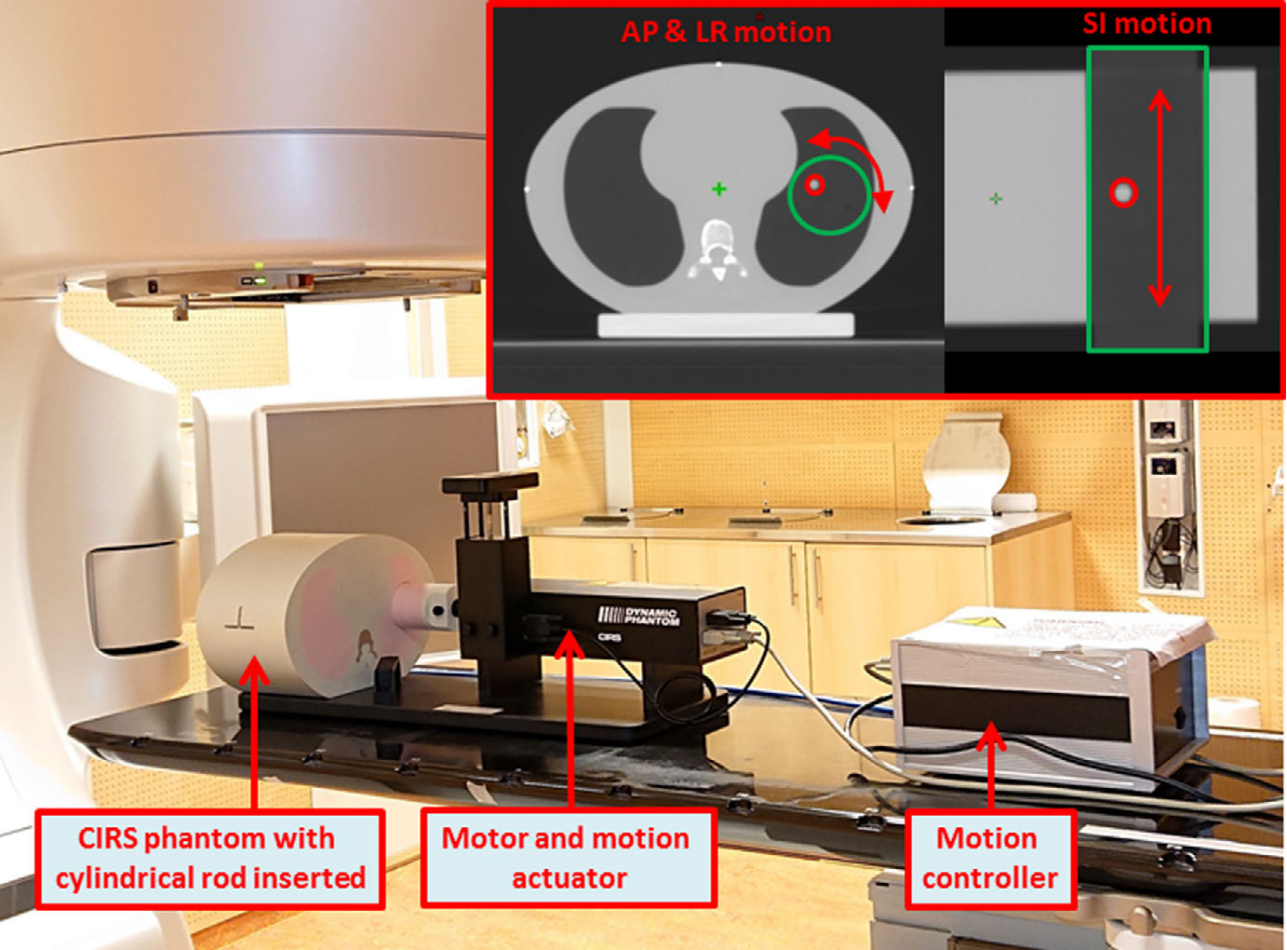
Measurement setup showing the CIRS phantom with its components on the treatment couch of a Varian TrueBeam linac. Tumor motion is simulated in the AP and LR directions by rotational motion of the rod, and SI tumor motion by translational motion of the rod, illustrated in axial and coronal images, respectively, of the CIRS phantom. The cylindrical rod is outlined in green color, while the off‐axis tumor insert is outlined in red.

A *CIRS Model 008A Dynamic Thorax Phantom* (CIRS Inc., VA, USA) was used in this study. This is a motion phantom suitable for investigating the impact of respiratory motion on both imaging and radiation treatment delivery for lung tumors. The phantom represents an average human thorax in shape, and contains anthropomorphic lungs and spine composed of tissue equivalent material. The linear attenuation of the lung equivalent material is within 3% of actual lung tissue attenuation for X‐ray energies between 50 keV and 15 MeV.^31^ The thorax phantom and its main components, illustrating the experimental setup on the Varian TrueBeam linac, are shown in Fig. 1. The phantom was initially aligned to the linac isocenter (left). The motor and motion actuator (middle) were physically connected to a cylindrical rod moving freely through one of the lung equivalent lobes of the phantom. The motion actuator allowed for precise translational motion of the rod along the couch axis, that is, the superior–inferior (SI) direction, as well as rotational motion around the same axis. The motion controller (right) was connected to the motion actuator as well as a computer, allowing remote control of the motion actuator, and thus actual motion of the rod.

The cylindrical rod is composed of the same lung tissue equivalent material, thereby making it more or less inseparable to the surrounding “lung” tissue. The rod may accommodate various inserts, such as tumor‐equivalent inserts of various sizes (with or without fitted ion chamber). These inserts are placed off‐axis in the cylindrical rod, enabling simulation of both anterior–posterior (AP) and left–right (LR) motion (by rotational motion of the rod) and SI motion (by translational motion along the couch axis). In this study, a spherical tumor insert with a diameter of 10 mm was used. Motion was controlled through a dedicated software, allowing independent control of the different motion axes. Motions in the AP and LR directions are to some extent dependent on each other (Fig. 1 **)**, as rotational motion of the rod will induce simultaneous AP and LR motion. However, by modifying the start angle of the rotational motion, the relative amplitudes of AP and LR motions were modified.

### 2.2 Motion patterns

The phantom software includes a number of built‐in motion curves. Users may also import patient‐specific curves. One may choose different motion curves — with different cycle periods and amplitudes — for different motion axes, allowing simulation of complex 3D tumor *motion patterns*. SI motion is limited to ± 25 mm, while AP and LR motion is limited to ± 5 mm. According to the manufacturer, motion accuracy is within 0.1 mm.^31^ The minimum cycle period is 1 sec, while there is no upper limit. Motion curves loop a specified number of times, and may be started and stopped at any point.

**Fig. 2:**
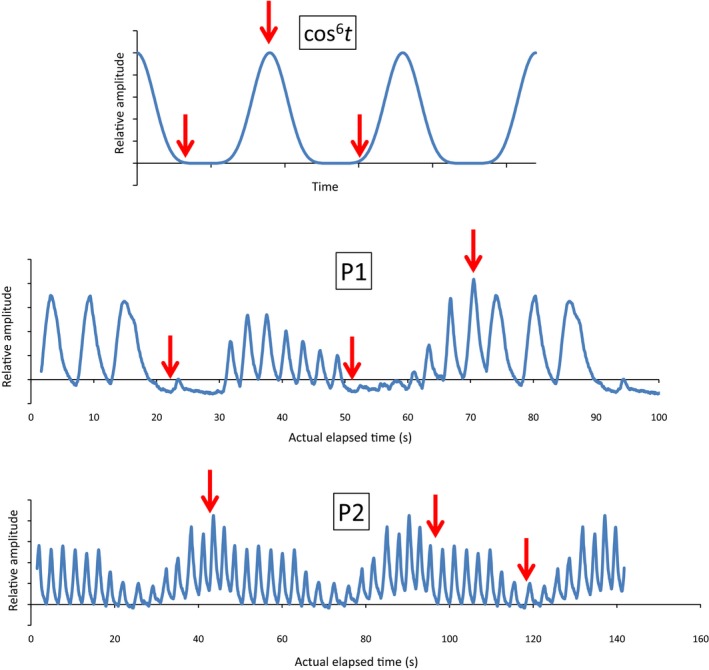
Motion curves used in the current study: built‐in *cos^6^t* curve (upper panel) and patient‐specific curves (middle and lower panel). Arrows indicate the points where beam‐on was initiated.

In this study, both built‐in and imported patient‐specific motion curves were used. The built‐in *cos^6^t* curve simulates a respiratory motion where the exhale phase is slightly longer than the inhale phase (Fig. 2, upper panel). The patient‐specific curves *P1* and *P2* (Fig. 2, middle and lower panel) were recorded in our clinic with the Varian TrueBeam Respiratory Gating System (Varian Medical Systems, CA, USA) during two different treatment sessions for the same patient, treated with stereotactic lung radiotherapy. The *P1* and *P2* curves were modified in such a way that a 70‐second and a 45‐second segment of the recording, respectively, repeated itself. The *absolute amplitudes* from the recordings were disregarded, as they represent chest wall surrogate motion — not tumor motion. The actual *cycle times* from the recordings, however, were regarded as actual respiration periods. The resulting motion patterns that were used during CT scanning and treatment delivery are summarized in Table 1. Amplitudes for irregular curves (*P1* and *P2*) refer to the *maximum* amplitudes.

**TABLE 1:** Motion patterns used in the current study during CT scanning and treatment delivery.

Motion pattern	Axis	Amplitude (mm)	Curve	Cycle period (s)
**A**	SI	5	cos^6^ *t*	4
	AP			
	LR			
**B**	SI	5	cos^6^ *t*	7
	AP			
	LR			
**C**	SI	20	cos^6^ *t*	4
	AP	5		
	LR			
**D**	SI	20	cos^6^ *t*	7
	AP	5		
	LR			
**E**	SI	10	P1	Actual
	AP	0	*N/A*	*N/A*
	LR			
**F**	SI	10	P2	Actual
	AP	0	*N/A*	*N/A*
	LR			
**G**	SI	20	P2	Actual
	AP	5	P1	
	LR			

### 2.3 CT scanning

A GE LightSpeed RT 16‐channel CT scanner (GE Healthcare, IL, USA) equipped with the Varian RPM gating system was used for all CT acquisitions. Motion patterns **A**, **C,** and **E** (Table 1) were chosen for CT scanning. With phantom motion paused, 3DCT scans using our standard lung protocol were performed to acquire image series of the (static) tumor in the maximum *inhale* and *exhale* positions, respectively, for all three motion patterns. Furthermore, 4DCT CINE scans were performed using our standard 4DCT lung protocol, and 10 phase bins, for each of the motion patterns A, C, and E. Scan acquisitions were started at random positions in the motion cycle. The *Average Intensity Projection* (AIP), *Maximum Intensity Projection* (MIP — for contouring purposes, not dose calculation), and the *mid‐ventilation phase* (midV) were reconstructed from all three 4DCT scans using GE dedicated software. The *midV* reconstruction was defined as the 30% phase bin (where 50% represents maximum *exhale* and 0%/100% represents maximum *inhale*). All image series were reconstructed with a slice thickness of 2.5 mm, corresponding to our current clinical practice.

### 2.4 Contouring

All contours were generated in the Varian Eclipse treatment planning system. Appropriate window width and window level for contouring the tumor insert was found to be 1 HU/−500 HU (Hounsfield units) — based on the fact that this gave the closest agreement between the actual insert size and the resulting contour. This approach is similar to the one described by Clements et al*.* 32 For all three MIP reconstructions, *iGTVs* — defined as the volume encompassing the GTV when taking motion into account — were generated using the above‐mentioned approach. Technically, iGTV contours were generated using the *thresholding* option with lower and upper limits of –500 HU and 2000 HU, respectively. The three iGTV contours (different sizes due to different motion amplitudes) were copied from their respective MIP reconstruction to their corresponding exhale, inhale, AIP, and midV image series generated by motion patterns A, C, and E. This resulted in four image reconstructions sharing the same iGTV, for each of the motion patterns A, C, and E — a total of 12 series. Thus, for each iGTV, there was an *exhale* image series, an *inhale* image series, an *AIP* image series, and a *midV* image series. iGTVs were denoted according to the motion pattern from which they were generated; *iGTV_A_, iGTV_C_*, and *iGTV_E_*. All iGTVs were contoured as “High accuracy structures.”

Clinical target volumes (CTVs) were generated by expanding the iGTVs by 5 mm, whereas PTVs were generated by expanding the CTVs by another 5 mm. This corresponds to our institution’s practice for stereotactic lung treatments while it is slightly smaller than our current standard margins for curative, fractionated lung treatments.

Lungs, spinal canal, heart, and body outline contours were also generated. Three dummy “OARs” were generated to tentatively produce a higher degree of modulation during the optimization process.

Interestingly, the iGTV generated from motion pattern **E**, *iGTV_E_*, did not fully encompass the *inhale* phases. This means that our 4DCT protocol was not able to image the entire tumor motion, even with an actual patient specific motion curve and a fairly realistic amplitude. We decided to keep this underestimated iGTV due to its relevance in a clinical scenario.

### 2.5 Treatment planning

Treatment plans were generated in the Varian Eclipse treatment planning system, using the AAA 13.6.23 algorithm. All plans (except two static plans) were VMAT plans — tentatively highly modulated — planned for a Varian TrueBeam linac. VMAT plans were planned with two semi arcs, and optimized using the PO 13.6.23 algorithm with heterogeneity correction enabled. Fractionation schemes were 2 Gy × 33 (normofractionated lung RT) and 15 Gy × 3 (highly hypofractionated lung SBRT). Plans with the same fractionation scheme were optimized using identical optimization parameters.

For *iGTV*_A_ and *iGTV_C_*, plans were generated with the exhale phase, the inhale phase, the AIP reconstruction, and the midV reconstruction as planning CTs, respectively — for both fractionation schemes. For *iGTV_E_*, plans were generated with the exhale phase, the AIP reconstruction, and the midV reconstruction as planning CTs, also for both fractionation schemes. In addition, for the latter iGTV, a static, non‐modulated plan with the exhale phase as the planning CT was generated for both schemes. This resulted in a total of 12 treatment plans for each fractionation scheme. Plan characteristics, in good agreement with our clinical practice for both ordinary fractionated and stereotactic plans, are summarized in Table 2.

**TABLE 2:** Plan characteristics for both high and low doses per fraction.

2 Gy/fraction (n = 12)		15 Gy/fraction (n = 12)	
Energy	6 MV	Energy	6 MV FFF
CTV D_98%_, *lowest* (%)	96.4	Point min. PTV, *lowest* (%)	95.3
PTV D_98%_, *lowest* (%)	87.0	Point max. PTV, *lowest* (%)	149.2
Point max, *highest* (%)	107.8	Point max. PTV, *highest* (%)	155.0
Mean CTV dose, *lowest* (%)	99.8	Mean CTV dose, *lowest* (%)	136.2
Mean CTV dose, *highest* (%)	100.2	Mean CTV dose, *highest* (%)	140.7
Calculation grid (mm)	2.5	Calculation grid (mm)	2.5
Collimator (arc 1/arc 2)	30°/330°	Collimator (arc 1/arc 2)	30°/330°
Normalization	100% median dose to CTV	Normalization	Compromise between 100% minimum and 150% maximum dose to PTV

CTV, Clinical target volumes; PTV, planning target volume.

**Fig. 3:**
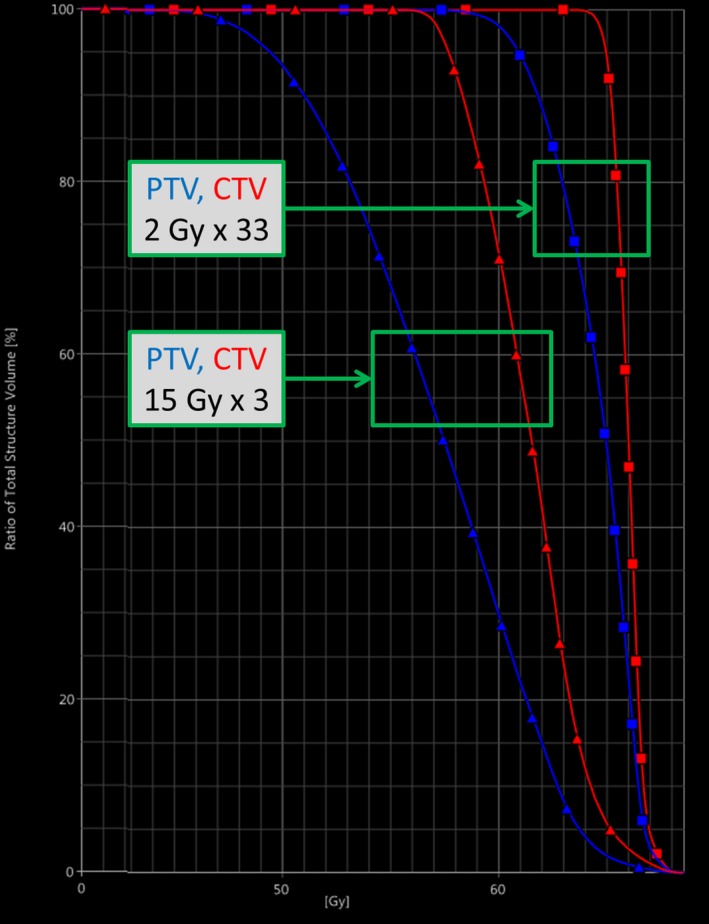
Typical CTV and PTV dose–volume histograms (DVHs) for both normofractionated and hypofractionated treatment plans. CTV, Clinical target volumes; PTV, planning target volume.

For *fractionated* lung radiotherapy with a curative intent, a total dose of 66 Gy in 2 Gy fractions is most often prescribed in our clinic. Plans are normalized such that 100% dose covers 50% of the target volume (median dose to the CTV). For all 2 Gy plans in the current study, the CTV D_98%_ was above 1.9 Gy (95% of the prescribed dose), and the maximum point dose was below 108% — both meeting our current clinical goals. For *stereotactic* radiotherapy in the lung, a dose of 15 Gy × 3 covering the PTV is commonly prescribed, depending on anatomy and nearby OARs. The minimum and maximum doses to the PTV are often a compromise between adequate target coverage and acceptable maximum dose. Thus, we neither have a strict 100% PTV D_min_ constraint nor a strict 150% D_max_ constraint. All stereotactic plans in the current study had a PTV D_min_ above 95% and a PTV D_max_ below 155%, as shown in Table 2. Typical CTV and PTV dose–volume histograms (DVHs) for fractionated and stereotactic plans, respectively, are shown in Fig. 3.

### 2.6 Measurements

A PTW *PinPoint* ionization chamber (PTW, Freiburg, Germany) with an active volume of 0.015 cm^3^ was inserted into the middle of the tumor insert, and used for all measurements in this study. Prior to plan delivery, linearity of dose and dose rate using the PinPoint chamber was assessed. For both 6 MV and 6 MV FFF, 100 MU and 300 MU were delivered to the phantom without motion, with chamber inserted. Furthermore, 100 MU was delivered at 100 MU/min and 600 MU/min (6 MV), and at 600 MU/min and 1400 MU/min (6 MV FFF).

The CIRS phantom was positioned using cone‐beam CT (CBCT) prior to plan delivery at the TrueBeam linac, ensuring precise coincidence of planned isocenter and physical linac isocenter. For plan delivery with the same motion pattern and the same amplitude at both CT and treatment delivery, the CBCT was matched “body outline to body outline,” and thus *treatment*‐iGTV to *planning*‐iGTV. For plan delivery with a different motion pattern at treatment delivery compared to CT — that is, different amplitude or motion curve — the CBCT was matched “exhale to exhale,” corresponding to a realistic clinical situation.^32^ Only translational couch corrections were performed.

Static reference measurements — that is, plan delivery without tumor motion — were performed with the tumor insert in the maximum *exhale* (cranial) position for all plans. Then, measurements were performed running the various motion patterns to assess possible impact of complex tumor motion on relative accumulated dose. Each measurement done with tumor motion was compared to the mean of the static reference measurements. For every plan, dynamic measurements were performed during the same session as static reference measurements, to account for day‐to‐day variations in machine output.

For each motion pattern, plan delivery (and measurement) was initiated at three different points on the motion curves, marked by arrows in Fig. 2, simulating the random nature of treatment delivery to patients breathing freely.

All static reference measurements, as well as the first measurement with tumor motion for every plan, were performed at least twice. A total of 143 and 128 plan deliveries were measured for 6 MV and 6 MV FFF, respectively.

The mean measured dose deviation per plan was calculated by averaging 7–15 measurements performed with the various plans.

**Fig. 4:**
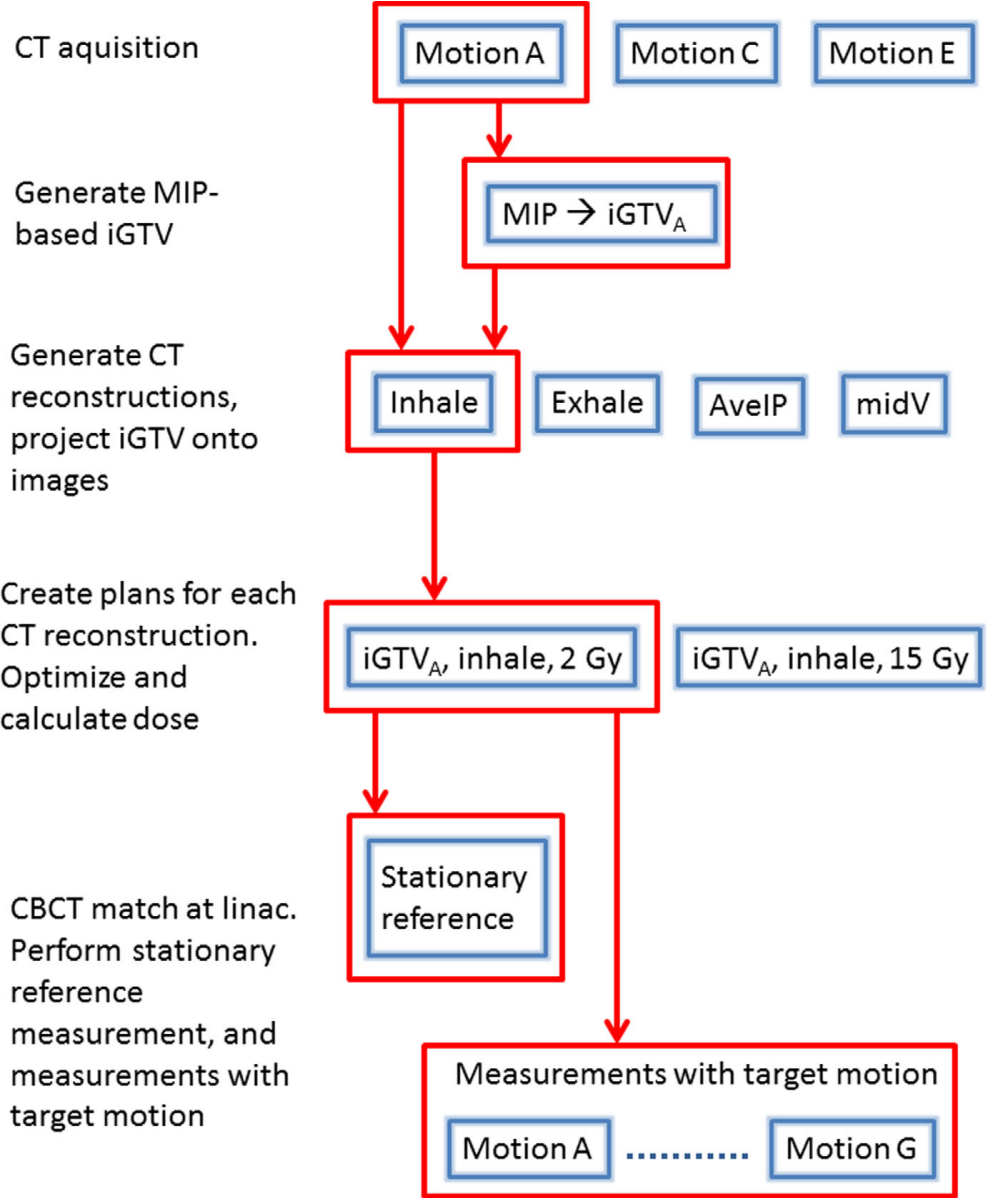
Simplified overview of the workflow used in the current study. One of the experimental tracks is highlighted.

A simplified, schematic overview of the experimental design is shown in Fig. 4. Only one “track” of the experiment is highlighted in the illustration — motion *A*, the *inhale* CT reconstruction and the *2 Gy* plan.

### 2.7 Plan complexity metrics

Plan complexity and homogeneity were assessed using various metrics. Possible correlations between measured dose deviations and the modulation complexity score applied to VMAT (MCSv), MLC leaf travel (LT), the average change in dose rate per control point (ΔDR/cp), monitor units pr. Gy (MU/Gy), and homogeneity index for the PTV (HI) were investigated.^22^, ^25^, ^33^, ^34^ A lower MCSv indicates a plan with more modulation, while a MCSv of 1 indicates no modulation, that is, a static, open field. HI was defined as $HI=\frac{D2\%-D98\%}{D50\%}$ . The MCSv, LT, and ΔDR/cp were obtained using an in‐house developed Eclipse script. For comparison, 10 randomly selected patient treatment plans were selected for each of the fractionation schemes. The average MCSv, LT, ΔDR/cp, and HI for the patient plans were compared to the corresponding values obtained in the current study.

### 2.8 Statistical analysis

When analyzing motion‐induced dose deviations, absolute values of deviations were evaluated. The non‐parametric Mann–Whitney test was used to analyze differences between dose deviations for 2 Gy and 15 Gy fractions, for every CT reconstruction. The same test method was used to analyze potential differences in dose deviations between the different CT reconstructions, separately for 2 Gy and 15 Gy fractions. SPSS software (SPSS, NY, USA) was used for statistical calculations. Statistical significance was considered at *P* < 0.05; however, Bonferroni correction was used for multiple testing.

Possible correlations between measured dose deviations and plan complexity metrics were assessed using the coefficient of determination, *R^2^*. Correlation analysis was done using Microsoft Excel 2010.

## 3 Results

Dose linearity and dose rate linearity measurements yielded negligible differences in accumulated dose for different doses and dose rates, both for 6 MV and 6 MV FFF.

Maximum dose deviations between plan delivery to the moving tumor and delivery to the stationary tumor are shown in Table 3. Numbers are “worst case” for each motion pattern, based on at least three measurements (at least one for each starting point on the motion curves, shown in Fig. 2). The largest motion‐induced dose deviations were 7.8% and 4.5% for 2 Gy and 15 Gy fractions, respectively. The largest observed dose differences from one fraction to another, both delivered to the moving tumor with the same plan, were −16.0% (iGTV_C_, midV) and 4.8% (iGTV_C_, exhale) for 2 Gy and 15 Gy fractions, respectively.

**TABLE 3:** Relative dose difference for plan delivery to a moving tumor, compared to plan delivery to a static tumor, for all iGTVs and all motions patterns. Numbers are "worst case" for each motion pattern, based on at least three measurements (corresponding to three starting points on the motion curves).

iGTV	CT recon. for optimization and calculation	2 Gy × 1 – 6 MV		15 Gy × 1 – 6 MV FFF		
		Motion during delivery		Motion during delivery		
		**A**	**B**	**A**	**B**	
iGTV_A_	Exhale	1.1%	2.7%	−0.9%	−1.5%	
	Inhale	2.1%	2.6%	−0.9%	−1.2%	
	AIP	2.9%	−2.1%	−1.9%	−1.1%	
	midV	1.9%	3.1%	−2.5%	−3.2%	

		**C**	**D**	**C**	**D**	**G**
iGTV_C_	Exhale	3.3%	2.5%	−0.7%	−1.5%	4.5%
	Inhale	0.7%	−4.1%	2.0%	2.3%	
	AIP	3.4%	1.6%	2.4%	2.5%	
	midV	5.9%	7.8%	3.1%	3.8%	

		**E**	**F**	**E**	**F**	
iGTV_E_	Exhale	−2.3%	−3.7%	2.5%	1.7%	
	AIP	−1.6%	−1.3%	−1.6%	−1.9%	
	midV	2.8%	1.2%	−1.0%	1.1%	
	Exhale, static fields	−0.2%	−0.1%	−0.1%	−0.3%	

AIP, Average Intensity Projection.

The mean measured dose deviations (absolute values) for each of the four CT reconstructions are shown in Table 4. The values were compared with each other pair wise, using the Mann–Whitney test with Bonferroni correction. The mean measured dose deviation for 2 Gy plans optimized with the midV CT reconstruction was significantly larger compared to measurements performed with all the three other CT reconstructions (*P* < 0.01). For 15 Gy plans, no significant differences were found between the various CT reconstructions (*P* > 0.4).

**TABLE 4:** Measured dose deviations for the various CT reconstructions used during plan optimization, for 2 Gy and 15 Gy fractions, respectively.

	2 Gy	15 Gy
Exhale	1.4%	1.2%
AIP	1.4%	1.0%
Inhale	1.5%	1.1%
midV	3.3%*	1.3%

The Mann–Whitney test including Bonferroni correction showed significantly larger dose deviation for 2 Gy plans optimized with the midV reconstruction(*) compared to the three other CT reconstructions (*P* < 0.01).

AIP, Average Intensity Projection.

For each CT reconstruction used during optimization, differences between measured dose deviations for the 2 Gy and 15 Gy fractionation schemes were calculated, and only had statistical significance for the midV reconstruction (*P* = 0.002).

Assuming equal probability per fraction for plan delivery to start at each of the three different points on the motion curves, the total motion‐induced dose deviation after 33 fractions of 2 Gy may be roughly estimated. For all 2 Gy plans and all measurements, the estimated summed dose deviation after 33 fractions were within 2%. For the three‐fraction SBRT regimen, a summed dose deviation is less meaningful, as the dose deviations per fraction are less likely to cancel each other out over only a few fractions.

**Fig. 5:**
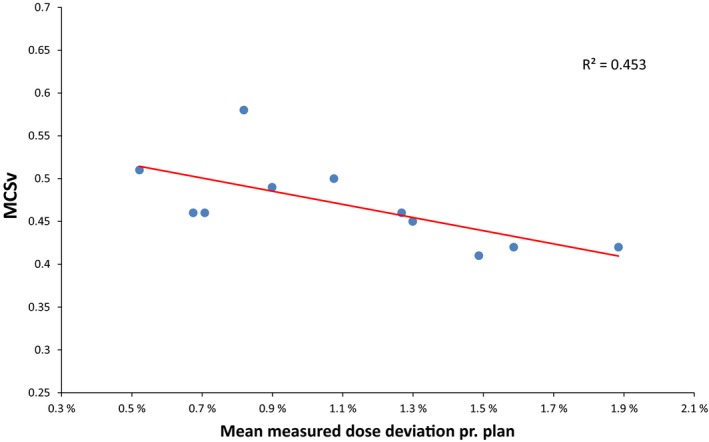
Mean measured motion‐induced dose deviations for 15 Gy plans vs. MCSv for each plan.

No correlation was found between measured dose deviations and *HI, LT, *Δ*DR/cp*, or *MU/Gy* for either fractionation schemes*.* For 2 Gy fractions, no correlation was found between measured dose deviations and *MCSv*; however, for 15 Gy fractions, a moderate correlation (*R^2^ = *0.45, *P* = 0.02) was found between the *MCSv* and the mean measured dose deviation for each treatment plan, as shown in Fig. 5. The slope of the fitted line in Fig. 5 indicates larger motion‐induced dose deviations for more modulated plans.

The 2 Gy plans in the current study had statistically significant higher *MCSv*, lower *LT*, lower Δ*DR/cp*, and higher *HI* compared to the patient treatment plans (*P* < 0.01). The 15 Gy plans, compared to 15 Gy patient plans, had statistically significant lower HI, lower *MCSv* but also lower *LT* (*P* < 0.01). Δ*DR/cp* was not different between patient plans and plans in the current study for 15 Gy fractions. It should be noted that most 15 Gy plans, both patient plans and plans in the current study, were delivered at maximum dose rate, thereby having a Δ*DR/cp* = 0.

## 4 Discussion

In the current study, we measured relative, motion‐induced dose deviations during delivery of both conventionally fractionated and hypofractionated VMAT plans. Relative dose deviations were obtained by comparing measurements performed with a stationary target and a target moving with a wide range of amplitudes and motion patterns.

Ong et al*.* 19, 20 investigated possible interplay effects when delivering VMAT SBRT plans to moving lung tumors both with and without flattening filter‐free (FFF) beams. Radiochromic film measurements with 15 and 30 mm tumor inserts, moving in a sinusoidal pattern, were performed using the QUASAR phantom (Modus Medical Devices Inc., Canada). For some of the plan deliveries, irradiation was initiated at three different points in the respiration cycle, as we did for all our plans in the current study. Amplitudes in the range 8–30 mm were used, some of which were based on actual tumor excursion measurements. In the latter study, motion‐induced dose deviations of up to 8.1% after one single fraction were reported — which is considerably higher than those found for the hypofractionated plans in the current study, perhaps influenced by larger motion amplitudes than those used in our study. In addition, 2D measurements using radiochromic film are more likely to reveal larger dose deviations around the target borders than a centrally located ion chamber.

The largest dose deviation measured in our study was for a single 2 Gy fraction (7.8%). The mean dose deviation per CT reconstruction and amplitude, over all measurements, was also always larger for 2 Gy plans than for 15 Gy plans. A possible explanation for this is that 15 Gy plans take more time to be delivered — meaning that possible interplay effects are more likely to cancel out due to more respiration cycles during beam‐on time. However, statistically significant differences between 2 Gy and 15 Gy plan delivery were only found for plans optimized with the midV CT reconstruction. A 2010 paper^18^ reported delivery of 2 Gy plans to a thorax phantom using various delivery techniques, including VMAT. They used a tumor model moving in 3D with patient‐specific motion patterns, and amplitudes comparable to the current study. Maximum motion‐induced dose differences between two single fractions of up to 16% were reported — which is in accordance with our results for 2 Gy fractions. However, the authors — as in the current study and previous works — report that the summed deviation after delivery of 30 fractions is typically within 2%, most likely due to averaging effects after many fractions.

In this study, the largest “worst‐case” motion‐induced dose deviations for 2 Gy and 15 Gy plans were — not surprisingly — found for measurements performed with the largest maximum SI motion amplitude (motion patterns *D* and *G*, maximum SI amplitudes of 20 mm). The largest mean deviation — over all measurements — was also found for motion patterns with a maximum SI amplitude of 20 mm. Thereby, it seems that patient breathing amplitude could be used at treatment planning as a parameter to identify patients who might need larger PTV margins, gated treatment, and/or special immobilization — such as abdominal compression — restricting respiratory motion above a certain threshold. Given that most lung tumors exhibit SI excursions of 10 mm or below, measurements with motion patterns *A, B, E*, and *F* are most relevant to typical clinical situations. The “worst‐case” motion‐induced dose deviations, for both fractionation schemes, were below 4% for measurements using these motion patterns. This indicates relatively high robustness of the treatment plans to breathing motion in most cases.

For iGTV_E_, a static non‐modulated plan was delivered to the phantom while running motion patterns *E* and *F*. With no chance of interplay between MLC leaves and target motion, dose deviations compared to delivery to a stationary target were practically zero, thus demonstrating the superiority of static non‐modulated plans regarding robustness to target motion. The same might be true for other non‐modulated delivery techniques, such as dynamic conformal arcs (DCA), or if VMAT plans are optimized using algorithms specifically developed for achieving robustness to motion. Nonetheless, one might question the relevance of investigating non‐modulated plans, as the dose conformity and OAR sparing achieved with VMAT or IMRT plans by far outperforms non‐modulated plans.

Ideally, patients’ respiratory motion would be highly regular, and stable between CT scanning and treatment sessions, as well as between treatment sessions. Otherwise, the iGTV generated at CT scanning might not be representative for respiratory motion of the target at treatment. A recent study^35^ compared pre‐treatment 4DCT and cine‐MR scans with MR scans obtained during treatment for 20 patients (7 with thoracic and 13 with abdominal lesions). The aim of the study was to investigate the ability of pre‐treatment scans to reliably capture and predict SI and AP tumor motion over the entire treatment course. The authors found that differences between pre‐treatment amplitudes (both 4DCT and MR) and treatment amplitudes were not significant. In the current study, we used two patient‐specific respiration curves (Fig. 2), recorded during two treatment sessions for the same patient. The curves display highly irregular respiratory motion, and are quite different — showing that respiratory motion might differ substantially between treatment sessions, and possibly between CT and treatment. It should be noted, however, that the patient‐specific motion curves used in the current study were *surrogate* motions — obtained by recording respiratory chest motion. Although actual tumor displacement is not directly comparable to a surrogate motion, we assumed that observed chest motion patterns to some degree indicated the motion patterns of the tumor — when disregarding absolute amplitudes. For the 15 Gy fractionation scheme, we found the largest deviation between stationary measurement and dynamic measurement when delivering a plan using *iGTV_C_* while running motion pattern *G* (Table 3)*.* Motion patterns C and G had identical amplitudes, but very different motion curves (Table 1).

The iGTV generated from motion pattern *E, iGTV_E_*, was underestimated when compared to the actual target motion during CT scanning. This implies that the target, during treatment delivery, occasionally would be outside the volume expected to encompass the entire tumor motion. However, motion‐induced dose deviations for plans using iGTV_E_ were not particularly large, even when highly irregular motion patterns were applied during delivery. The “missing part” of the iGTV was in the caudal end — the inhale phase — where most tumors spend the least amount of time during a breathing cycle. Thus, the total time spent outside the (underestimated) iGTV constitutes a very small part of the total beam on‐time.

Our comparison of measured motion‐induced dose deviations for plans optimized with different CT reconstructions was only of statistical significance for the 2 Gy midV‐based plans. The *inhale* phase is normally considered the least frequent and least representative for most patients, and it was thus expected to observe substantial dose differences particularly in this phase. It is likely that a larger tumor equivalent volume than in the current study (Ø = 10 mm) yields different results in this regard. It should be noted that optimizing plans on two different CT reconstructions obviously generates two different treatment plans. This, in turn, means that differences in measured motion‐induced dose deviations might also be coincidental — since the two plans have different MLC motions. Furthermore, the current study did not investigate the agreement between *TPS dose calculation and measurement* with various CT reconstructions, as effects related to this are eliminated using the static measurement as reference — and the main aim was to quantify the impact of optimizing on different CT reconstructions on *robustness to target motion* during plan delivery. Previous studies^36^ have investigated the agreement between TPS *calculated* dose and *delivered* dose for SBRT VMAT plans, for various modifications of a phantom target volume. However, comparisons with a static reference measurement were not done. Nevertheless, a study aiming to investigate agreement between TPS calculated dose and measured dose should take into account possible limitations of the dose calculation algorithm. It has been shown that the AAA algorithm overestimates dose for heterogeneities in lung tissue.^37^, ^38^, ^39^ Since our measurements were performed with a centrally located ion chamber, this possible underdosage around edges of lung heterogeneities was not revealed.

Some authors have reported correlations between dosimetric accuracy and various plan complexity metrics,^22^, ^23^, ^24^, ^26^ while others did not find such correlation.^25^ In the current study, we investigated possible correlations between measured motion‐induced dose deviations and plan complexity metrics for highly hypofractionated SBRT lung plans as well as normofractionated lung radiotherapy plans. A moderate correlation was found between MCSv for average measured dose deviations for the 15 Gy plans, but not for the 2 Gy plans. The slope of the fitted line in Fig. 5 indicates lower robustness to target motion for plans with lower MCSv, meaning higher motion‐induced dose deviations for more modulated plans — which is also expected. Although similar observations were expected for other plan complexity metrics, we did not find correlations between HI, LT, ΔDR/cp or MU/Gy, and measured dose deviations. If all other contributing factors besides plan modulation could be controlled and accounted for, stronger correlations might have been found. Such factors might be the finite mechanical precision of the linac, or setup uncertainty — and thereby accuracy of ion chamber positioning. Comparison of plan complexity metrics between the experimental 2 Gy plans in this study and randomly selected 2 Gy patient treatment plans yielded more modulated patient treatment plans. However, since no clear correlation was found, we cannot conclude that dose deviations due to target motion are higher for the patient treatment plans. Similarly, even though the homogeneity metric was found to be significantly different for the experimental plans compared to patient plans, it is not possible to conclude that this has an impact on robustness to target motion, based on the results of this study.

Villaggi et al^40^ evaluated other plan metrics such as *PI* (Plan Irregularity: deviations in field aperture shape compared to a circle) and *MIt* (Modulation Index total, which considers variations in gantry speed and acceleration, dose rate variation, etc.) Evaluating additional plan complexity metrics in the current study might have revealed stronger correlations between plan characteristics and the dosimetric impact of motion.

It seems there is a potential for further investigations on whether plan complexity metrics, along with the irregularity of respiratory patterns, may predict robustness of treatment plans to target motion.

A limitation of the current study is the use of only a 1‐cm‐diameter “tumor” sphere, which is more relevant to the SBRT setting than to the target volumes typically seen in standard, fractionated radiotherapy of lung cancer. One should also bear in mind the limitations of only measuring a single small volume in the center of the moving target. The results reported by previous authors^17^ indicate that larger dose deviations are seen in the borders of the target along the direction of the motion, rather than the central part. Measuring 2D or 3D dose distributions would probably reveal such effects.

In the current study, we initiated plan delivery at three different points on the respiration curves (Fig. 2), as was done by, for example, Ong et al*.* 20 Using more starting points, like Jiang et al*.* 14 who used eight, might have yielded larger motion‐induced dose deviations.

## 5 Conclusion

For single 2 Gy fractions, maximum dose differences of 7.8% between static and dynamic measurements were observed. These effects appear to be attributable to interplay between MLC leaves and tumor motion. From one 2 Gy fraction to the next, a maximum dose difference of 16% was noted. This may not be clinically important, as the summed deviation — due to respiratory motion — after delivery of 33 fractions is probably less than 2%. For single 15 Gy fractions, the maximum observed motion‐induced dose difference was 4.5%. As the central tumor dose in lung SBRT is typically 30–50% higher than the prescribed PTV minimum dose, this deviation might not be clinically important — provided that the PTV periphery dose coverage is adequate. Non‐modulated plans with static MLCs showed superior robustness to target motion during delivery. Only minor overall differences, especially for 15 Gy fractions, were found when comparing measured dose differences for plans optimized with different CT reconstructions. Moderate correlations between MCSv and measured dose deviations were found for 15 Gy SBRT treatment plans. Correlations between other plan complexity metrics and measured dose deviations were not found.

## Conflict of Interest

No conflicts of interest.
